# Culture dependent and independent approaches reveal the role of specific bacteria in human skin aging

**DOI:** 10.1002/imo2.26

**Published:** 2024-09-10

**Authors:** Jing‐jing Xia, Qian Zhong, Zhi‐ming Li, Qing‐zhen Wei, Liu‐yi‐qi Jiang, Cheng Duan, Hui‐jue Jia, Yi‐mei Tan, Lian‐yi Han, Jean Krutmann, Jiucun Wang, Xiao Liu

**Affiliations:** ^1^ State Key Laboratory of Genetic Engineering, Collaborative Innovation Center for Genetics and Development, School of Life Sciences and Human Phenome Institute Fudan University Shanghai China; ^2^ Greater Bay Area Institute of Precision Medicine (Guangzhou), School of Life Sciences Fudan University Guangzhou China; ^3^ Department of Skin & Cosmetic Research Shanghai Skin Disease Hospital Shanghai China; ^4^ IUF ‐ Leibniz Research Institute for Environmental Medicine Düsseldorf Germany; ^5^ Research Unit of Dissecting the Population Genetics and Developing New Technologies for Treatment and Prevention of Dermatological Diseases (2019RU058) Chinese Academy of Medical Sciences Shanghai China; ^6^ Shenzhen International Graduate School Tsinghua University Shenzhen China

**Keywords:** *Cutibacterium acnes*, fungi, keratinocyte, *Moraxella osloensis*, skin aging, skin microbiome, shotgun metagenomic sequencing

## Abstract

Skin aging is a dynamic process involving a spectrum of phenotypic changes, making it an attractive model for studying microbiome‐phenotype interactions. Therefore, 822 facial microbial samples and 14 skin phenotypes from corresponding areas were assessed in a Chinese cohort. Porphyrins and the chronological age exhibited the most significant microbial variability. We further profiled the dynamics of the skin microbiome associated with age and aging phenotypes. Using a multiple linear regression model, we predicted premature/delayed aging‐related microbial species, mainly *Moraxella osloensis* and *Cutibacterium acnes*. We also validated the biological functions of the host‐microbe interactions in vitro. *Moraxella osloensis* isolated from healthy skin regulates collagen metabolism and extracellular matrix assembly, and promotes cell senescence in human keratinocytes and fibroblasts, making it potentially applicable in the development of antiaging interventions.

## Highlights

- Age‐and aging phenotype‐related skin microbiome dynamics were profiled using a shotgun metagenomic sequencing data set.
- *Moraxella osloensis* and *Cutibacterium acnes* were predicted to be premature/delayed aging‐related microbial species using machine learning modeling.
- *Moraxella osloensis* isolated from healthy skin regulates collagen metabolism and extracellular matrix assembly in vitro.
- Age had a relatively minor influence on the variation in the fungal community compared to that in the bacterial community. Age‐related fungal taxa were analyzed in this study.

## Graphical abstract

In total, 822 facial microbial samples and 14 skin phenotypes were assessed in a Chinese cohort. We profiled skin microbiome dynamics associated with age and skin aging phenotypes. Using machine learning‐based skin aging models, we predicted the microbial species associated with premature or delayed aging. Functional experiments with skin aging‐related microbes isolated from healthy human skin have shown that *Moraxella osloensis* regulates collagen metabolism and extracellular matrix assembly in human keratinocytes and fibroblasts.

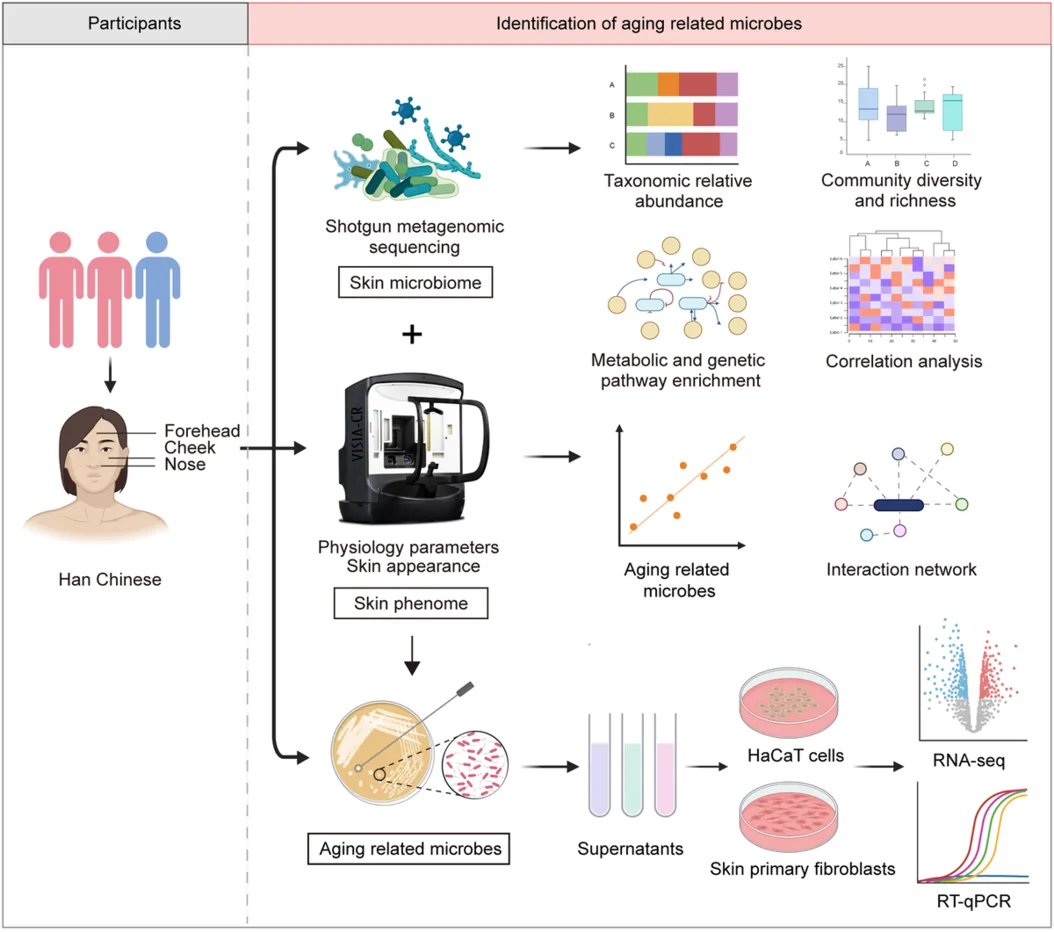

## 1 INTRODUCTION

The skin is colonized by millions of diverse microorganisms, including bacteria, fungi, and viruses, collectively referred to as the human skin microbiome [1]. These commensals are important for maintaining skin homeostasis, and disruption of the skin microbiome is associated with various clinical conditions in some of the most prevalent skin diseases [2].

Even in healthy humans, the skin microbiome is highly individualized and dynamic [3, 4]. Numerous variables influence skin bacterial communities, including host factors (such as race, sex, and age) and environmental factors due to occupation or lifestyle (e.g., diet, hygiene, skin products, and medication) or other exposures (e.g., climate, geographical location, pollution, ultraviolet, and other radiation) [5, 6]. Nevertheless, from the perspective of classic ecology, given that the skin is perceived as an ecosystem [7], the selection pressures shaping the ecosystem can be simplified into three main elements: (1) resource availability (presence of nutrients), (2) environmental conditions (temperature and geographical access), and (3) biological factors (occupation of ecological niches through self‐adaptation or microbe–microbe interactions) [8, 9]. Previous evidence also strongly suggests that the physical and chemical landscapes of distinct niches primarily drive local microbial composition (niche selection) [3, 7, 10, 11]. In turn, the growth and metabolism of these microinhabitants modify local niches, such as the pH or moisture state, and reshape the host skin phenotypes [7, 9, 12]. However, understanding the skin microbiome–phenotype interaction remains ambiguous and requires systemic characterization.

Skin aging is a dynamic process with a spectrum of age‐related phenotypic changes in appearance and function, making it an ideal model for studying microbiome–phenotype interactions. Although numerous studies already confirmed the impact of chronological age in skin bacterial communities [13, 14, 15, 16], the understanding of cross‐kingdom microbiome variation with skin aging remains minimal. However, this is not a trivial task. Although, in most conditions, age correlates well with skin aging phenotypes, or vice versa, one's perceived age may deviate largely from the actual chronological age, because skin aging is a complex process that combines chronological and extrinsic aging [17].

**Figure 1:**
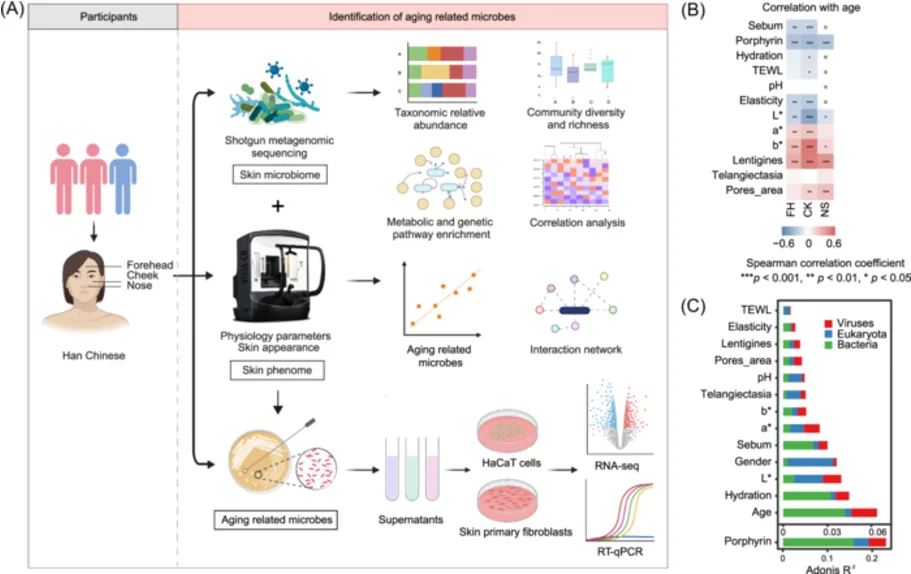
Study workflow, correlation analyses, and microbial composition influences. (A) The overall workflow of this study. (B) Spearman's correlation between phenotype parameters and age. FH, forehead. CK, cheek. NS, the back of the nose. L* = skin darkness/lightness; a* = erythema; b* = tanning. An increase in L* value indicates a transition from black to white, while a* values range from green to red, and b* values shift from blue to yellow. Blue, negative correlation. “o” denotes missing data on transepidermal water loss (TEWL), hydration, sebum, pH, and elasticity due to anatomical constraints in the back of the nose. The color bar represents correlation values. The significance levels in the Spearman correlation are: *p < 0.05; **p < 0.01; ***p < 0.001. (C) Bar charts illustrating the effect size of variables on bacterial, fungal, and viral compositions. Variables found to be significant with species were shown. Adonis R^2^ values represent the fractions of microbial composition variation explained by the variables. Different colors represent different kingdoms. Partially created with BioRender.com.

To explore cross‐kingdom microbiome interactions with skin phenotypes, 294 healthy individuals were recruited from Shanghai, which is by far the largest sample size for skin metagenomic profiling in the Chinese population. Skin microbiomes from three facial sites, the forehead (FH), cheek (CK), and back of the nose (NS), were collected. A total of 822 samples were assessed using shotgun metagenomic sequencing, which allowed for a more precise survey across all kingdoms (bacteria, fungi, and viruses). Multiple potential host variables, including age, sex, and 12 skin phenotypes, were cross‐analyzed to elucidate host–microbiota interactions in healthy Chinese individuals. In contrast to previous studies, this study takes this step further; in addition to addressing the aging microbiome correlation, we used two‐dimensional (2D) cultures to validate these associations. The general workflow of the study is shown in Figure 1A.

## 2 RESULTS

### Chronological age plays a crucial role in skin phenotypes and explains a large skin bacterial variability

The skin microbiome and phenome (a collection of skin traits) of the corresponding sampling areas were assessed (Figure 1A). Owing to anatomical restrictions, some parameters from the NS were not collected. As expected, age had a central impact on skin appearance and physiology (Figure 1A and Figure S1A–C). The scores for lentigines, telangiectasia, and pore area increased with age, whereas those for sebum content, porphyrins, hydration, and skin elasticity declined with age (Figure 1B), consistent with our knowledge of skin aging. In addition, skin color became more dull (L*), yellowish (b*), and reddish (a*) with increasing age, which is consistent with previous findings [18, 19] (Figure 1B). Notably, pH was not significantly associated with age, and transepidermal water loss (TEWL) reflecting skin barrier function unexpectedly decreased with age. These inconsistencies may further imply the existence of a deviation in skin aging from chronological age. These associations were generally of a high degree of consistency in the three facial sites.

We then assessed skin microbial variations associated with different host features using permutational multivariate analysis of variance (PERMANOVA). Fourteen factors were significantly correlated with the skin microbiota (adjusted *p* < 0.05, Figure 1C). Overall, porphyrin explained the highest microbiome variance, especially in the bacterial community, which is consistent with previous findings in North American volunteers [10]. This was followed by age and hydration level. Notably, for the fungal community, age very marginally affected variation, whereas sex had a prominent effect on variation (Figure 1C). Notably, many skin phenotypes, such as skin color (L*, a*), telangiectasia, pH, and TEWL explained more variability in the fungal community than in the bacterial community. Variability in the viral community, which is commonly considered a transient member of the skin microbiota [20], was also associated with porphyrin levels, age, and other skin traits (adjusted *p* < 0.05, Figure 1C). Co‐inertia analysis revealed a significant relationship between the viral and bacterial communities (permutation tests, *p* < 0.01), and the abundance of bacteria and their corresponding bacteriophages were highly correlated (Figure S2A,B), suggesting that across‐kingdom microbe‐microbe interactions underlie the community balance of the ecosystem.

### Age‐related skin microbiome dynamics

**Figure 2:**
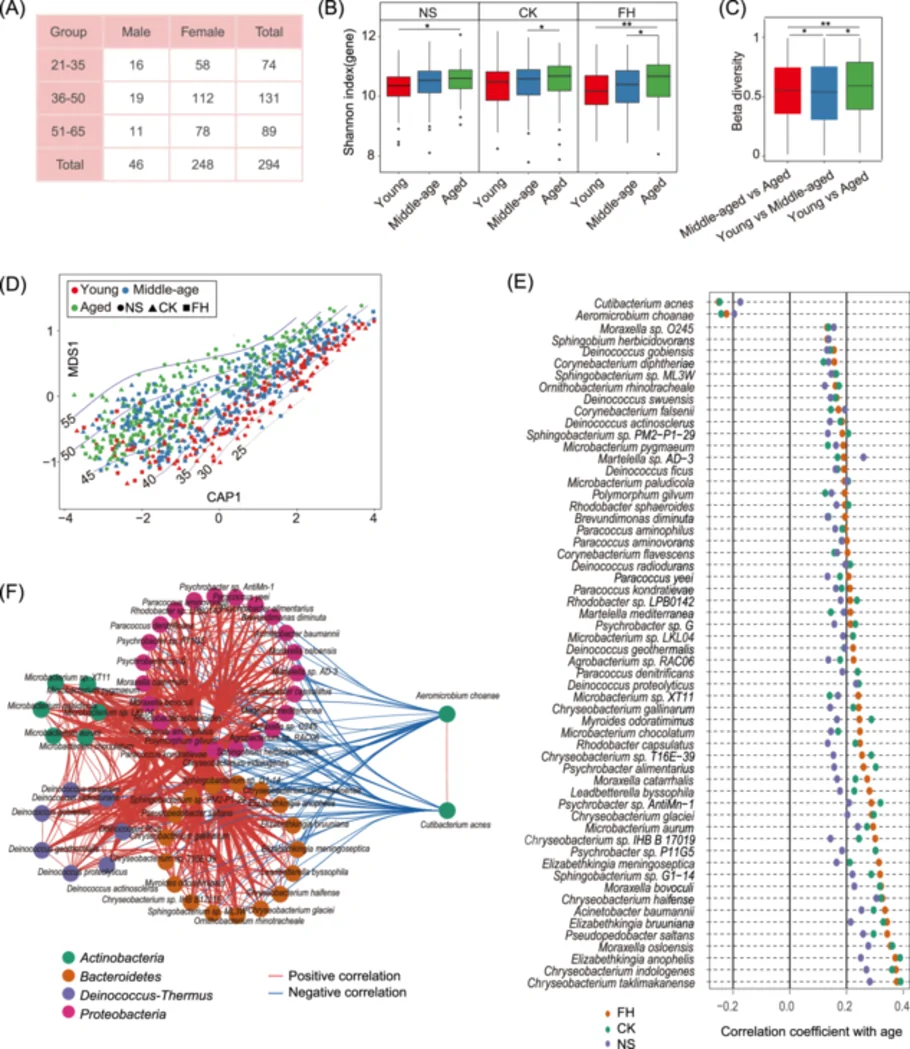
Dynamic changes of bacterial community during aging. (A) Different age groups. Young group (21–35, *N* = 74), middle‐aged group (36–50, *N* = 131), and old group (above 50, *N* = 89). (B) Alpha diversity of the bacterial community increased slightly during aging. Wilcox test is used to determine significance. (C) The beta diversity of the bacterial community was different between the three age groups. Wilcox test is used to determine significance. (D) Distance‐based redundancy analysis (dbRDA) depicting the variations of bacterial community composition between different age groups. Each point represents a single sample. Colors represent age groups. Shapes represent different sites. Lines represent different ages. (E) Associations between bacterial taxa and age. Colors represent three sites: Green, CK; Orange, FH; Purple, NS. (F) Co‐occurrence network of age‐related species. The nodes represent age‐related species. The colors of nodes represent the phylum. Red lines represent positive correlations. Blue lines represent negative correlations.

In addition to porphyrin, age was the most influential factor affecting bacterial variance (Figure 1C). To identify age‐associated species, we explored bacterial dynamics across different age groups: young (20–35 years, *N* = 74), middle‐aged (36–50 years, *N* = 131), and old (above 50 years, *N* = 89) (Figure 2A).

Overall, bacterial diversities from all tested facial sites increased with age (Figure 2B), which is consistent with previous findings [21, 22, 23, 24, 25, 26]. In addition, beta diversity between the groups showed that the greater the age difference, the more significant the change in the skin bacterial microbiome (Figure 2C). Distance‐based redundancy analysis demonstrated a clear separation between the microbial species in the different age groups (Figure 2D). A comparison of the bacterial species across different age groups identified 58 age‐associated bacterial species (Figure 2E). While most species increased with age, especially *Moraxella* spp. (mainly *Moraxella osloensis*), *Chryseobacterium* spp. (mainly *Chryseobacterium taklimakanense*), *Elizabethkingia* spp., and *Paracoccus* spp., two species progressively decreased with age, namely *Cutibacterium acnes* and *Aeromicrobium choanae* (Figure 2E). *A. choanae* was been associated with the skin microbiome in other studies. We found this species in very low abundance, but *Aeromicrobium phoceense*, another species of the same genus, has been isolated from the skin [27]. Notably, these two species showed a synergistic effect that was positively correlated with each other and negatively correlated with other age‐increasing species (Figure 2F). In addition to skin bacteria, we found three viruses associated with age (Spearman's *p* < 0.05): *Betapapillomavirus 3*, *Staphylococcus virus phiETA*, and *Streptococcus phage IPP18* (Figure S2C).

### The skin microbiome is highly correlated with skin aging features

Skin aging is a dynamic process involving a series of phenotypic changes in the appearance and physiological parameters. These phenotypes can reflect niche conditions, such as pH and hydration, or are the ultimate manifestations of microbial metabolic activity [9].

**Figure 3:**
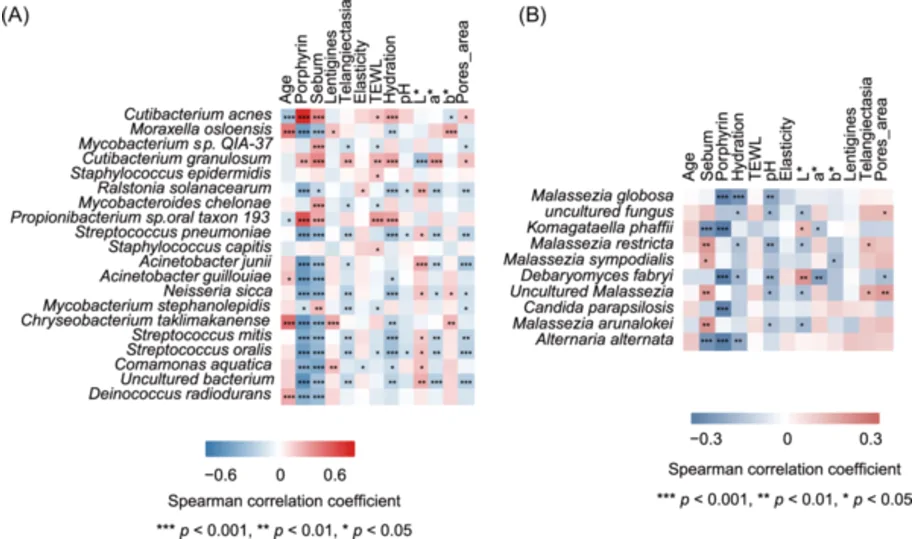
Correlation between bacteria, fungi, and phenotype parameters. Heatmap of the Spearman's correlation between the top 20 bacteria (A) and top 10 fungi (B) in abundance and skin phenotype parameters on the forehead. Blue, negative correlation; Red, positive correlation. The significance levels in the Spearman correlation are: *p < 0.05; **p < 0.01; ***p < 0.001.

To specify phenotype‐related microbial dynamics, we performed a cross‐kingdom Spearman's correlation analysis between the microbiome and aging phenotypes (Figure 3). Of the 20 most abundant bacterial species, *C. acnes* and *M. osloensis* showed opposite trends. The abundance of *C. acnes* was positively correlated with the levels of porphyrin, sebum, TEWL, hydration, and pore area, but negatively correlated with age and yellowish skin color (b*). In contrast, *M. osloensis* was prone to aging traits, such as reduced hydration, increased lentigines, and increased yellowish skin color (Figure 3A). Another well‐known skin commensal, *Staphylococcus epidermidis* showed a similar tendency towards *C. acnes*‐associated patterns; however, except for TEWL, the other associations were not significant (Figure 3A and Figure S3).

Although fungi account for a minority of the microbial community, their cell sizes are typically a hundred‐fold larger than those of bacterial cells, and many fungi are considered influential in shaping host phenotypes through their unique metabolism [28]. Therefore, we further evaluated the skin phenotype‐associated fungal members. Many fungal species were associated with distinct skin traits (Figure 3B). Notably, many *Malassezia* species positively correlated with skin sebum levels, which is consistent with their lipophilic properties.

### Machine learning‐based skin‐age modeling to locate premature‐aging/delayed‐aging‐related microbial species

Although chronological age is central to most skin aging phenotypes in population, one's perceived age, or following called “skin‐age” may deviate from their chronological age. An applied skin‐age algorithm was developed based on machine learning to assess the perceived age. The algorithm was trained on 200,000 images, using chronological age as the training tag, and fine‐tuned in the perceived age cohort (*N* = 5768). The correlation coefficient between the model‐predicted value and mean facial age perceived by the investigators reached 0.97.

**Figure 4:**
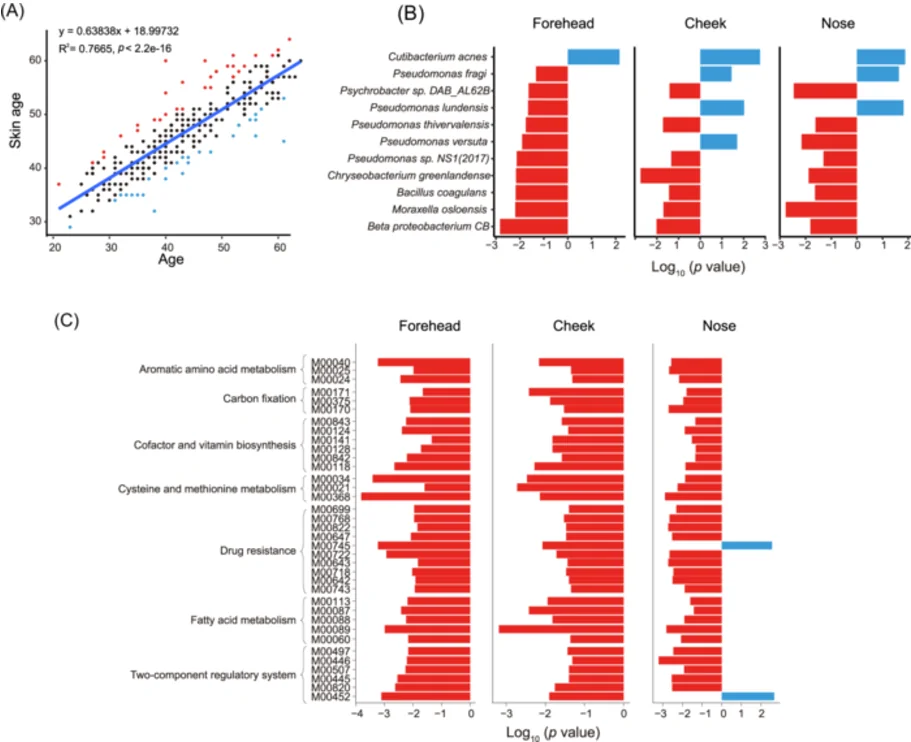
The differences in microbial composition and functions between premature‐aging group and delayed‐aging group. (A) Multiple linear regression (MLR) model was used to predict skin‐age. Red, belonging to premature‐aging group; blue, belonging to delayed‐aging group. (B) The differences in microbial composition between the young skin group and the elderly skin group on the forehead, cheek, and nose. Red, bacteria that enriched in the elderly skin group. Blue, bacteria that enriched in young skin group. (C) Features of bacterial functions between premature‐aging and delayed‐aging group on the forehead, cheek, and nose. Bar‐chart showing the relative abundance of differential Kyoto Encyclopedia of Genes and Genomes (KEGG)‐modules. Red, modules enriched in the premature‐aging group. Blue, modules enriched in the delayed‐aging group.

A multiple linear regression (MLR) model was used to predict skin age. The residual standard error (RAE) achieved using the MLR model configuration was 3.639 years, with 0.7665 of *R* ^2^. To identify aging/antiaging related microbial species, we filtered individuals whose Δage (difference between predicted and chronological age) was greater than RAE and divided them further into premature‐aging (*n* = 30) and delayed‐aging (*n* = 27) groups (Figure 4A). We sequentially compared the differences in skin microbial composition between the two groups. Bacterial species, such as *M. osloensis*, *Bacillus coagulans*, *Chryseobacterium greenlandense*, and *Pseudomonas thivervalensis* were more abundant in the premature aging group. In contrast, *C. acnes* was enriched in the delayed‐aging group (Figure 4B). To explore the potential function of aging‐related species, we compared bacterial KEGG KOs between groups. Notably, the microbiome of the premature aging group exhibited significant gene module enrichment in the two‐component regulatory systems, resistance, and drug efflux transporters/pumps (Figure 4C). The enrichment of resistance genes may be due to frequent or prolonged exposure to antibiotics, suggesting that the skin microbiome has experienced selective pressure, leading to an increase in antibiotic‐resistant strains. Two‐component systems are signal transduction systems in bacteria that respond to adaptive environmental changes, such as pH, temperature, or the presence of antibiotics [29]. Enrichment in the premature aging group suggests that the skin microbiome may need to adjust its physiological state more frequently to adapt to harsher or changing microenvironments. In contrast, the microbiome from the delayed‐aging group was enriched in the phosphotransferase system responsible for transporting carbohydrates [30] and central carbohydrate metabolism, which may represent active growth and energy demands from these commensals.

### *M. osloensis* regulates collagen metabolism and extracellular matrix assembly in human keratinocyte and fibroblast cells

*M. osloensis* and *C. acnes*, also known as cutotype‐determinant species in Chinese skin [31], were significantly correlated with age and skin aging phenotypes (Figures 2E, 3A, and 4B). While *C. acnes* negatively correlated with age and aging traits, such as increased sebum/porphyrin production, higher elasticity and hydration levels, and fewer lentigines, *M. osloensis* exhibited the opposite correlation and was prone to aging traits. We also assessed the phenotypic association with *S. epidermidis*, a well‐known dominant skin commensal, and found that it had a very weak correlation with skin phenotype (Figure S3).

**Figure 5:**
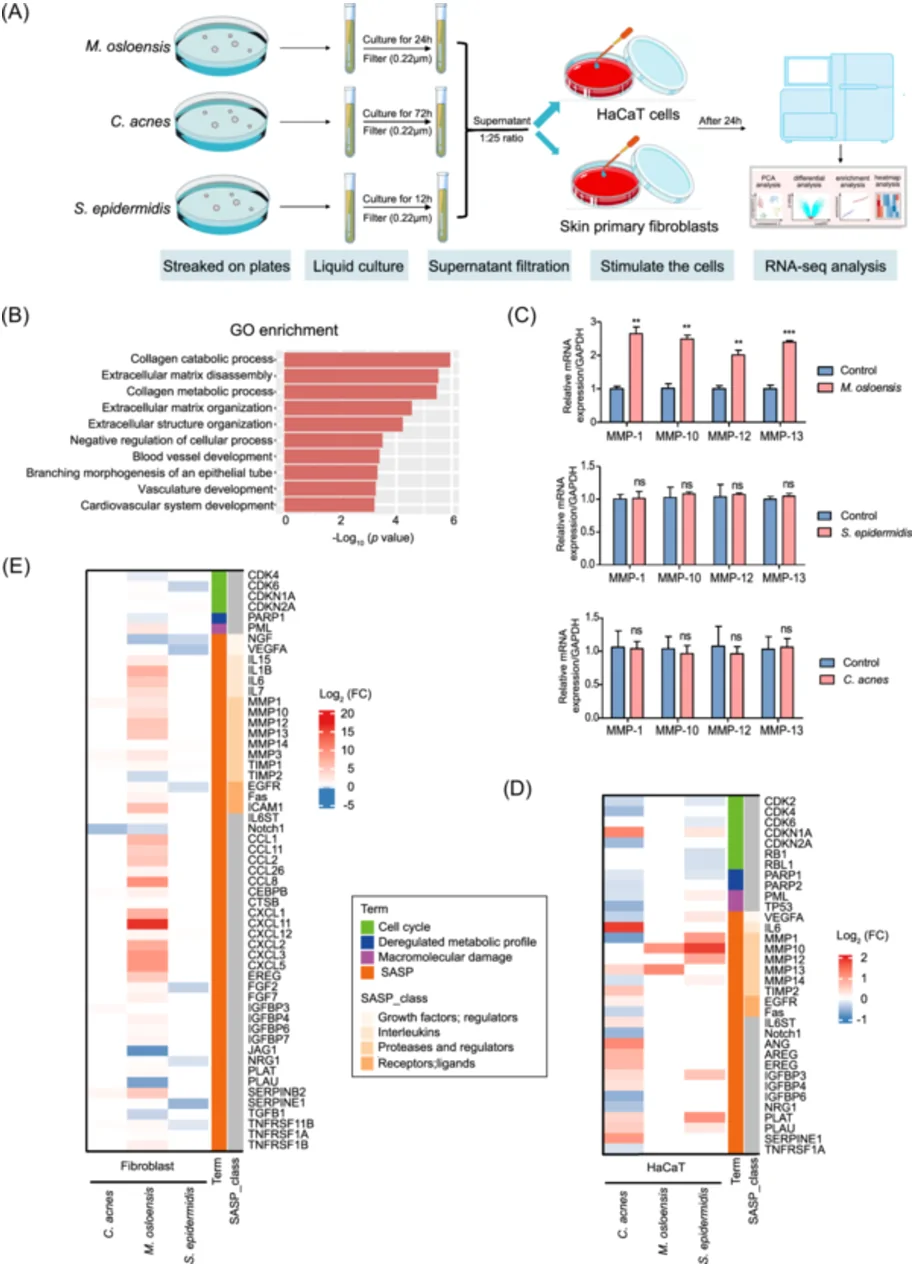
Genes expressed by keratinocytes and fibroblasts cocultured with three skin bacteria. (A) Scheme of skin cells stimulated by bacterial supernatant. RNA‐seq analysis of HaCaT cells and primary dermal fibroblast upon 24 h of stimulation with supernatant from *Moraxella osloensis*, *Cutibacterium acnes*, and *Staphylococcus epidermidis* grown in R2A, RCM, or LB medium, at OD_600_ 0.8–1.0 (*n* = 3). The cells stimulated by the bacterial culture medium (R2A, RCM, or LB) served as the control group. (B) Gene Ontology (GO) enrichment showing the top 10 enrichment pathways of HaCaT cells treated with bacterial supernatant from *M. osloensis* (*p* < 0.05). (C) Gene expression analysis of several matrix metalloproteinases (*MMPs*) in HaCaT cells upon 24 h of stimulation with supernatants of *M. osloensis, C. acnes*, and *S. epidermidis*, individually, using quantitative polymerase chain reaction (qPCR) (*p < 0.05; **p < 0.01; ***p < 0.001. data pooled from *n* = 3 independent experiments). (D) and (E) are heatmaps illustrating the expression changes of aging related genes in HaCaT cells (D) and fibroblasts (E), each treated separately with the supernatants of three diffrerent microbial species (adjusted *p* < 0.05). The gray‐colored bars indicate that the corresponding genes have no further subclassification.

The correlation between skin traits and bacteria might be associated with skin niches‐driven bacterial variance, or vice versa. To further explore the host‐microbe interactions potentially underlying these associations, we performed RNA‐seq analysis of human epidermal keratinocyte (HaCaT) and primary dermal fibroblasts with supernatants from the above commensals [32] (Figure 5A). Gene Ontology (GO) enrichment analysis of HaCaT cells showed clear enrichment of differentially expressed genes (DEGs) regulating the collagen catabolic process, extracellular matrix disassembly, and collagen metabolic process upon treatment with *M. osloensis*‐conditioned medium (Figure 5B). Quantitative real‐time polymerase chain reaction (RT‐qPCR) results confirmed that the RNA expression of many matrix metalloproteinases (*MMPs*) from *M. osloensis* supernatant treatment group was significantly upregulated (Figures 5C,D), consistent with the association between *MMP*s and skin aging, as these enzymes are known to be major contributors to collagen degradation and skin wrinkling [33, 34]. The results also revealed a marked accumulation of DEGs implicated in cell senescence according to Senequest [35], a database of cell senescence (http://Senequest.net), when human dermal fibroblasts were incubated with the supernatant from *M. osloensis*, but not *C. acnes* or *S. epidermidis*. In *M. osloensis‐*conditioned medium, fibroblast cells were characterized by altered gene expression during cell cycle arrest, senescence‐associated secretory phenotype, macromolecular damage, and a deregulated metabolic profile (Figure 5E). These cellular changes may have deleterious effects on the tissue microenvironment and contribute to a wide range of age‐related pathologies [36]. We showed that the *MMP* expression induced by *S. epidermidis* and *C. acnes* was not as substantial as that induced by *M. osloensis*, especially in primary dermal fibroblasts. This suggests a potentially unique role for *M. osloensis* in influencing collagen metabolism and extracellular matrix assembly in skin cell models.

### Age‐related skin fungal dynamics

**Figure 6:**
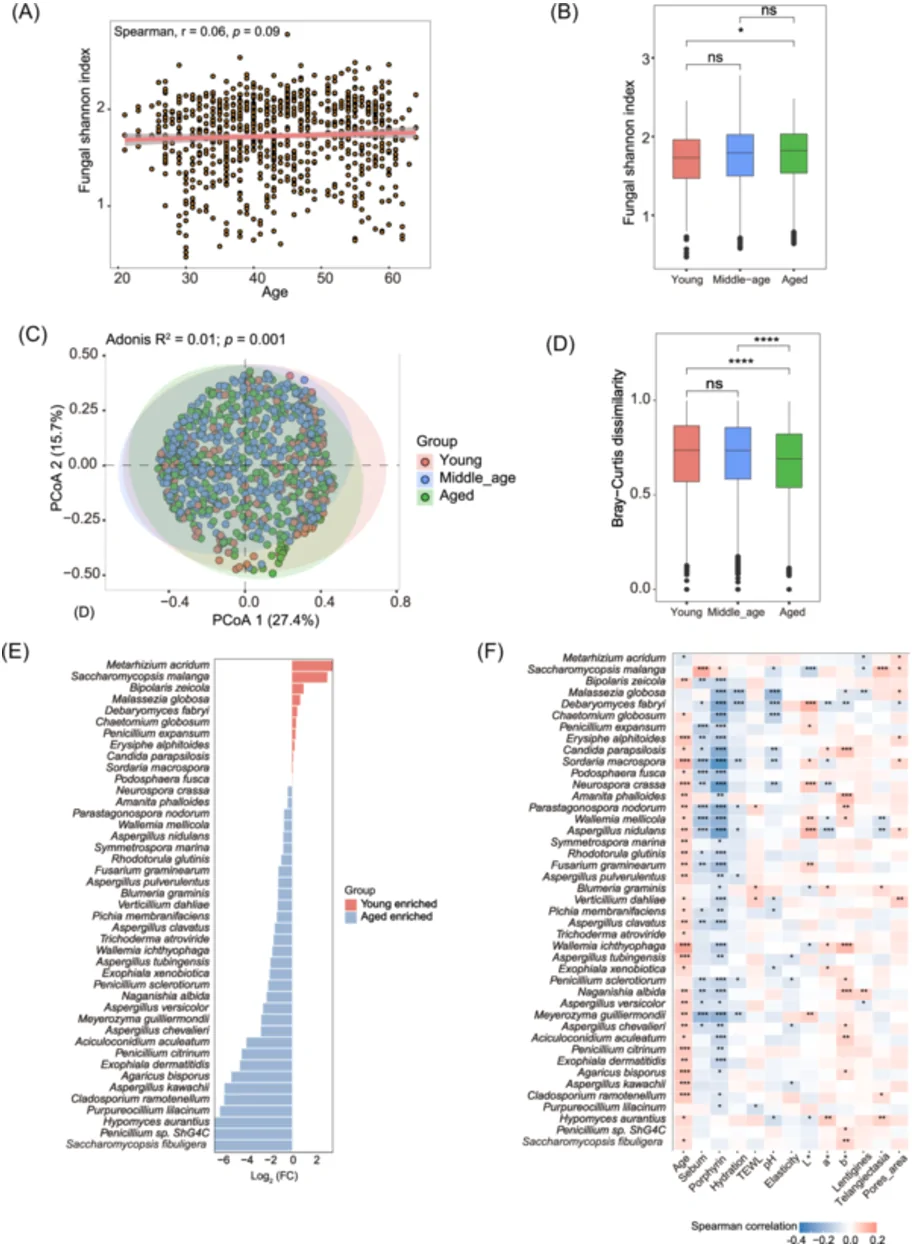
Dynamic changes of fungal community in three age groups. (A) Spearman correlation analysis between alpha diversity and age in fungal communities. (B) Box plots comparing fungal alpha diversity between three age groups: young (20–35, *N* = 74), middle‐aged (36–50, *N* = 131), and aged (>50, *N* = 89). The significance was determined using the Wilcoxon test. (C) Principal co‐ordinates analysis (PCoA) of Bray–Curtis distances for the fungal communities in each individual across the age groups at the species level. Samples in each age group are clustered by 95% confidence ellipses. The PERMANOVA test is used to determine significance. (D) The beta diversity of the fungal community between the three age groups. Community composition similarity was measured based on the Bray‐Curtis distance between two samples within age group at the species level. (E) Bar chart of differences in fungi composition between the young and aged group, determined by Wilcoxon rank sum test. Red bar, fungi that enriched in the young group. Blue bar, fungi that enriched in the aged group. (F) Heatmap of Spearman correlation analysis between the differential fungi in (E) and 13 host factors. Statistical significance is indicated as follows: ***p < 0.001; **p < 0.01; *p < 0.05.

Shotgun metagenomic data allowed us to study the dynamics of fungal communities. Compared with its significant impact on the bacterial community, age had a minor effect on the variation in the fungal community (Figure 1C). No significant correlation was found between age and the fungal Shannon index (*p* = 0.09) (Figure 6A). We then explored fungal dynamics across age groups. Fungal α‐diversity in the aged group was higher than in the young group (Figure 6B). This is consistent with a previous study on 65 individuals from Sardinia using the internal transcribed spacer 1 (ITS1) gene amplicon sequencing method [37] and a study on healthy Korean women [23]. Principal coordinate analysis (PCoA) based on Bray–Curtis distance revealed that the grouping of fungal communities by age was statistically significant (*p* = 0.001) (Figure 6C). The Kruskal–Wallis test revealed that the fungal community compositional variations within the young and middle‐aged groups were significantly larger than those in the aged group (*p* < 0.05) (Figure 6D).

To identify age‐related fungal taxa, Wilcoxon rank‐sum test was performed (Figure 6E). Eleven fungal species were significantly enriched in the young group, and 32 were significantly enriched in the aged group. For example, *Malassezia globosa* and *Debaryomyces fabryi* were enriched in the younger age group. Subsequently, we conducted a Spearman correlation analysis to investigate the relationship between the differential fungi and skin traits (Figure 6F). The results showed that most of the differentially expressed fungi were positively associated with age and negatively associated with porphyrin and sebum levels.

## 3 DISCUSSION

The driving force behind the establishment of various human commensal systems remains unclear. Whether it is a random process or involves selection, or which selection, if any, are the most concerning issues in the field. Previous studies have suggested that neutral drift with niche selection from the host can give rise to the biodiversity and biogeography of human microbiomes [11, 38]. Distinct skin niches have been proposed to drive the local microbial composition [3, 7, 10]. In turn, skin commensals are able to modify local physiology, such as pH or hydration, by converting host‐derived nutrients into acidic metabolites [9, 39, 40, 41, 42, 43].

Notably, in this study, we found that porphyrin, among all the measured variables, explained the highest microbial variability, which is consistent with the findings of a recent large‐cohort study of North American populations [10]. These results strongly suggest that, regardless of the ethnic population, porphyrin‐producing species play a central role in maintaining the stability of the facial skin ecosystem. Several *Cutibacterium* species, such as *Cutibacterium granulosum*, *Cutibacterium avidum*, and *Cutibacterium modestum* (previously, “*Propionibacterium humerusii*”) were able to produce porphyrin [44]; however, *C. acnes* is the main contributor [45, 46]. *C. acnes* is one of the most essential symbiotic microorganisms in the human skin, promoting the accumulation of oleic acid in the skin and metabolizing fatty acids or other oil components into propionic and acetic acid, thereby eliminating harmful microorganisms and protecting human skin [47]. This species is also prone to sebum‐rich environments associated with lower biodiversity. Given these results, we propose that *C. acnes* is the foundation species in the skin ecosystem, a species that controls the biological diversity of associated species, modulates critical ecosystem processes, and often has essential cultural value and resonance [48].

Skin aging is associated with changes in the cutaneous physiology, including interactions with the skin microbial community. Many *16S* rRNA gene surveys have reported the effects of age on skin microbiome composition in different ethnic populations. Despite substantial disparities in species in different populations, a higher alpha diversity/species richness of bacteria in the elderly was observed in both Asians [21, 49, 50, 51] and western Europeans [15]. Specifically, the aged population exhibited a substantial increase in the abundance of *Corynebacterium* but a decrease in *Cutibacterium* [15, 22]. This result contradicts the fact that higher biodiversity correlates with better eco‐stability (healthier skin), and decreased biodiversity is often linked to fragile stability and skin conditions. As the elderly endure more skin conditions, we propose that the increased diversity in the elderly is a passive consequence of the decline of the foundation species in the ecosystem, namely *C. acnes*. This is also consistent with the fact that skin sebum and porphyrin levels decrease during aging, and the decline in *C. acnes* can accommodate the survival of other species.

In this study, we found *C. acnes* was significantly correlated with age and skin‐aging phenotypes (Figures 2E, 3A, and 4B). *C. acnes* negatively correlated with age and aging traits, such as increased sebum/porphyrin production, higher elasticity and hydration levels, and fewer lentigines. Larson et al. similarly found *C. acnes* was more abundant in young adult skin, while older adult (aged ≥65) skin showed a substantial depletion of *C. acnes* [52]. The decline in *C. acnes* among elderly individuals may be due to age‐related changes in skin physiology, such as decreased sebum production. The subsequent loss of *C. acnes* may create a suitable environment for opportunistic microbes to colonize and multiply, resulting in a more diverse microbial community. Compared to younger adults, the skin niche of the elderly is less selective, leading to increased instability and loss of immunity or nutrition selectivity [52]. In addition, Larson et al. revealed that the relative abundance of *C. acnes* was significantly associated with the Rockwood Frailty Index in the face, torso, antecubital fossa, and hands. This result prompted us to consider whether *C. acnes* plays an important role in human aging, frailty, and diseases associated with aging, which requires further investigation in the future.

A series of studies have also suggested other microbiome‐phenotype associations. For example, a study on the skin microbiome of Koreans found that *Lawsonella* was negatively correlated with skin moisture and brown spots, *Staphylococcus* and *Corynebacterium* were negatively correlated with the number of UV spots and positively correlated with TEWL, and *Staphylococcus aureus* was negatively correlated with skin moisture parameters [24]. However, most of these studies required direct evidence with functional validation. In this study, we characterized aging‐related species, such as *M. osloensis*. This species has been rarely studied and very few reports have indicated that it is a pathogen in immunocompromised adults [53]. However, this species was proven to be an essential member of the human skin commensals [54, 55] and emerged as the second most abundant species on the skin of the Han Chinese [31]. Here, we confirmed the association of *M. osloensis* with premature aging traits and further validated its capability to promote skin cell senescence, which explains the premature aging phenotype in the Chinese and other elderly populations [52].

Despite rapid progress being made towards deciphering the human virome, several roadblocks remain to be fully characterized and utilized [56]. Owing to methodological difficulties [57], the human skin virome is the least known community in the skin microbiome. It still needs to be determined whether the viruses are actually a normal part of the human skin flora or mutually benefit the host [57]. Numerous phages were found in our cohort, similar to a previous study that reported strong core phagosome presence in a North American population [20]. Notably, the abundances of bacteriophages and their corresponding bacteria were highly correlated. Our data further revealed a potential from phenome interaction for skin traits, such as porphyrins, age, skin color, pore area, and lentigines, which explained a certain level of virome variability. Given their low abundance and knowledge of the gut microbiome [58, 59], these virome‐phenotype associations are likely due to virus‐bacterial interactions.

Our study focused on the facial microbiome by specifically analyzing samples from the forehead, cheeks, and nose, which are the areas most exposed to environmental factors and are indicative of visible aging. While this approach provides critical insights into the changes in the skin microbiome with age, we acknowledge that it presents a limited view confined to the facial skin. Different body sites have unique microenvironments that may exhibit diverse responses to aging. Consequently, our findings, although significant, may not fully represent systemic changes across the entire skin microbiome. Future studies should consider larger samples to obtain a more comprehensive picture of the evolution of the skin microbiome throughout the aging process.

In this study, we present data from in vitro experiments that suggest a role for *M. osloensis* in modulating the expression of *MMP*s and potentially influencing skin aging. Although these findings provide valuable insights, we recognize the inherent limitations of in vitro studies and the importance of validating these results in more complex biological systems. The use of animal models would undoubtedly strengthen the evidence for our claims by allowing us to observe the effects of *M. osloensis* within an integrated system in which multiple cell types and systemic factors interact. Such models offer a more representative depiction of the skin aging process and the role of the microbiome within a living organism. However, owing to the constraints of our study design and resource availability, animal model experiments were not included in the current study. Despite this limitation, we believe that our in vitro findings provide a solid foundation for future studies. We highlighted the importance of in vivo experiments to confirm and extend our understanding of how *M. osloensis* and other skin microbiota contribute to the complex mechanisms of skin aging.

## 4 CONCLUSIONS

In this study, the interpretation of results from shotgun metagenomic sequencing, accompanied by culture‐dependent approaches, led to a better understanding of the composition, diversity, and variability of the human skin microbiota, even providing some relevant information about functional and mechanistic attributes. In the present study, we (i) profiled age/phenome‐related skin microbiome dynamics, (ii) predicted premature aging/delayed aging‐related microbial species, mainly *M. osloensis* and *C. acnes*, and (iii) validated the biological functions of some host‐microbe interactions in vitro.

Altogether, the current research has moved beyond the question of which microbes are present on the skin to assess the link between their function and outcome on the skin. We systematically characterized the skin microbiome‐phenome association network, especially the aging phenotypes. Furthermore, we validated some predicted associations with in vitro functional experiments, as a deep understanding of the molecular pathways underlying the aging process is critical in searching for products that target skin microbiota to improve skin phenotypes. Therefore, our study is important for designing interventional strategies for various age‐related skin conditions.

## 5 METHODS

### Participants and microbial collection

The study population and microbial sampling results were consistent with those described previously [31]. In short, 294 healthy participants (46 males and 248 females) aged 20–65 years were recruited from the general population in Shanghai from April to May 2017. Individuals with a history of skin diseases or antibiotic use within the past 6 months were excluded from the study. To maximize the skin microbial load, the participants were instructed to wash their faces with tap water and avoid applying any skincare or cosmetic products on the sampling day [60]. Samples were collected from the FH, CK, and NS. For DNA extraction, new sterile polyester fiber‐headed swabs, pre‐moistened in a solution of 0.15 M NaCl and 0.1% Tween 20 [61], were used to swab the specified site 40 times over an approximately 4 cm² area, ensuring firm pressure and rotational movement to thoroughly coat the swab. The swab heads were then broken off, placed in sterilized 1.5 mL centrifuge tubes, and stored at −80°C [3]. A total of 822 facial microbial samples were obtained.

### Skin phenotype assessment

Skin physiological parameters were measured in a controlled environment (temperature: 20 ± 1°C, relative humidity: 50 ± 5%) following a 30‐min acclimatization period for each participant. To minimize personnel error, the same investigator operated each device consistently throughout the study. Skin traits were determined in 294 healthy volunteers, including TEWL, hydration status, sebum, surface pH, and elasticity, which reflect the ecological niche environment and a series of skin appearance features, such as constitutive skin color (L*, a*, and b*), pore area, porphyrin, lentigines, and telangiectasia.

TEWL, a key indicator of skin barrier function [62], was assessed using a Vapometer® (Delfin Technologies Ltd). Skin hydration in the stratum corneum was evaluated using a D compact moisture meter (Delfin Technologies Ltd.). Sebum levels were quantified using a Sebumeter® SM815 (Courage & Khazaka electronic GmbH), with results expressed in μg/cm². The skin pH was determined using a Skin‐pH‐Meter (PH 900; Courage & Khazaka Electronic GmbH). Skin elasticity was measured using a Dual MPA 580 Cutometer (Courage & Khazaka Electronic GmbH). In this study, the parameter used to characterize the elasticity was R7. R7 = instantaneous elastic recovery/maximum suction depth of the suction phase (Ur/Uf), ranging from 0% to 100% [63]. The higher the value of R7, the more elastic the skin [64]. Skin color parameters (L*, a*, and b*), along with assessments of lentigines, telangiectasia, pore area, and porphyrin levels, were evaluated using ImageJ software, and images were captured using the VISIA‐CR system (Canfield Scientific Inc.). An increase in the L* value indicates a transition from black to white, a* values range from green to red, and b* values shift from blue to yellow. Porphyrins, lentigines, and telangiectasia were manually assessed using UV fluorescence, normal light, and RBX RED images, respectively. Images were scored independently by three trained scorers using a standardized protocol based on a photoreference scale. The scores range from 0 (hardly present) to 3 (severe). The final score for each parameter used for further analysis was the sum of the three scorers. Bacterial porphyrins have the potential to induce inflammation, and elevated levels have been associated with various inflammatory diseases in humans, including the prevalent skin condition, acne vulgaris [44]. Telangiectasia refers to the dilation of small blood vessels of varying diameters in the superficial dermis. These are frequently observed in individuals with fair skin, particularly in sun‐exposed regions of older adults [65]. Information on skincare frequency was gathered from the participants via a questionnaire, focusing primarily on the frequency of skincare routines; specific skincare products were not detailed.

### Acquisition and analysis of public metagenomic data

Metagenomic data of the skin microbiome were obtained from our previously published article [31]. Following methods described previously, we mapped the data to a 10.9 M skin microbial gene catalog [31] and calculated the relative abundance of bacteria in each sample and the relative abundance of KEGG functions for subsequent statistical analysis.

### Permutational multivariate analysis of variance

A permutational multivariate analysis of variance (PERMANOVA) was used to evaluate the impact of various covariates, including age, sex, physicochemical indices, and skin image data, on all profile types. This analysis was conducted using the vegan package in R, with 1000 permutations to derive the permuted *p* value.

### Correlation analysis

Spearman's correlation coefficients were computed for relationships between the relative abundance of the identified species and skin phenotypes or other species. A scaled heat map was constructed for the correlation matrix. Multiple omics correlations were visually presented using the Corrplot package in R (v3.5.3). FDR‐adjusted *p* values of less than 0.05 were used as the detection threshold for significance.

### Multivariate analysis

Multivariate statistical analyses (principal component analysis [PCA] and PCoA) were performed to assess the skin microbiome within individuals. PCA was performed on three facial sites, as previously described. PCoA was performed based on the Jensen–Shannon/Bray–Curtis distance on the skin fungal composition and functional profile using the ade4 package.

### Machine learning‐based skin‐age modeling

The skin‐age algorithm was developed by Meitu Eve based on the deep learning model MobileNet V3 to assess perceived age. The algorithm was trained on 200,000 images using chronological age as the training tag and fine‐tuned in the perceived age cohort (*N* = 5,768). The correlation coefficient between the model‐predicted value and mean facial age perceived by the investigators reached 0.97.

### Bacterial isolation

Five healthy volunteers with no visible acne on their faces and no recent topical antibiotic use were sampled to isolate three commensal skin bacteria (*M. osloensis*, *C. acnes*, and *S. epidermidis*). The volunteers ranged in age from 23 to 56 years old, with two males and three females: Subject 1 (female, 23 years old), Patient 2 (female, 24 years old), Patient 3 (female, 56 years old), Patient 4 (male, 27 years old), and Patient 5 (male, 30 years old).

Samples were taken from the FH and CK using swabs soaked in a solution of 0.15 M NaCl. The sampling region was swabbed 20 times. Facial swab specimens were sequentially used to inoculate R2A agar and Tryptic Soy Agar (TSA) with 5% sheep blood and placed under aerobic and anaerobic growth conditions. After incubating R2A agar for 48 h and TSA blood agar for 5 days in an anaerobic incubator, hundreds of colonies grew on both plates. From a literature search, we found that all three species form small grey to white round colonies on agar plates. Colonies were picked based on phenotypic diversity, followed by primary screening using specific primers *M. osloensis*‐F (5ʹ‐GGTGAAAGGGGGCGCAAGC‐3ʹ) and *M. osloensis*‐R (5ʹ‐GTGCTTA TTCTGCAGGTAACGTCTAATC‐3ʹ), *C. acnes*‐F (5ʹ‐GGATAACTTCAGGAAACTGGGG CT‐3ʹ) and *C. acnes*‐R (5ʹ‐TACCCATTACCGTCACTCACGC‐3ʹ), *S. epidermidis*‐F (5ʹ‐TCTTCAAGAAGCTCACTCAGAG‐3ʹ) and *S. epidermidis*‐R (5ʹ‐ ATTCCCACGCTAAC GATTTACG‐3ʹ). PCR samples that produced clear bands during gel electrophoresis were likely to be the target bacteria. The colonies were characterized by *16S* rRNA gene sequencing. *M. osloensis* was successfully isolated from the face of Patient 3 using nutrient‐poor R2A agar. In contrast, *C. acnes* was successfully isolated from the face of young Patient 2 using TSA blood agar. Additionally, *S. epidermidis* was isolated from the faces of almost every patient using R2A agar. Vigorously growing colonies of the three bacteria were selected for subsequent experimental validation.

### Bacterial growth conditions and supernatant preparation

*M. osloensis* were streaked on R2A plates and incubated overnight at 30°C. The following day, individual colonies of this strain were grown in liquid R2A medium at 30°C overnight, shaking at 220 Revolutions Per Minute (RPM). Cultures of activated strains were inoculated into fresh R2A medium (2%). After 24 h of further incubation (OD_600_ 0.8–1.0), bacteria were spun and culture supernatants were filtered twice using 0.22 μm Spin‐X centrifuge tube filters (Corning) [32]. The supernatants were stored at −80°C for the following experiments. The supernatants of *C. acnes* (RCM medium, 37°C, anaerobic) and *S. epidermidis* (LB medium, 37°C, aerobic, 220 rpm) were similarly collected. The difference is that their supernatants were collected 72 and 12 h after inoculation to reach the desired OD_600_.

### Cell culture

Normal human skin fibroblast cultures were established from punch biopsies obtained from healthy male donors who had undergone circumcision [66]. Briefly, skin specimens were immersed in normal saline and washed three times with phosphate‐buffered saline. The skin specimens were cut into 1 mm^2^ sections and laid onto the surface of a 6‐cm Petri dish (epidermis upward and dermis downward). Dulbecco's modified Eagle's medium (DMEM) supplemented with 10% (v/v) fetal bovine serum and 1% (v/v) penicillin‐streptomycin (Gibco) was added to each dish, and the medium was changed every 2–3 days. A dense outgrowth of cells appeared after 7–14 days and cells were passaged using 0.25% trypsin‐EDTA [67, 68]. Primary fibroblasts within five generations were used in all experiments. The immortalized HaCaT human keratinocyte cell line was purchased from the China Center for Type Culture Collection (Wuhan). All cells were incubated in standard incubators at 37°C with 5% CO_2_.

### Bacterial supernatant stimulation

HaCaT cells and normal skin primary fibroblasts were plated on 6 cm Petri dishes and grown to 80% confluence. The filtered supernatants of *M. osloensis, C. acnes*, and *S. epidermidis* were diluted in DMEM (1:25 ratio) and used to stimulate the cells for 24 h [32]. Cells stimulated with the bacterial culture medium (R2A, RCM, or LB) served as the control group.

### RNA‐seq

Total RNA was extracted from HaCaT cells and primary fibroblasts using TRIzol reagent (Thermo Fisher Scientific) in accordance with the manufacturer's guidelines. RNA integrity was evaluated using an RNA Nano 6000 Assay Kit on a Bioanalyzer 2100 system (Agilent Technologies). cDNA was synthesized using M‐MuLV Reverse Transcriptase and DNA Polymerase I. Library fragments were purified using the AMPure XP system (Beckman Coulter). PCR amplification was conducted using Phusion High‐Fidelity DNA polymerase, Universal PCR primers, and Index (X) Primer, followed by purification of the PCR products using the AMPure XP system. Library quality was verified using an Agilent Bioanalyzer 2100 system. Index‐coded samples were clustered on a cBot Cluster Generation System using TruSeq PE Cluster Kit v3‐cBot‐HS (Illumina). Clusters were subsequently sequenced on an Illumina NovaSeq platform, producing 150 bp paired‐end reads.

The initial raw data in fastq format were processed using custom Perl scripts. During this step, clean reads were obtained by filtering sequences with adapters, poly‐N regions, and low‐quality reads. The reference genome index was constructed using Hisat2 v2.0.5, and clean paired‐end reads were aligned to the reference genome using the same software. Read counts mapped to each gene were quantified using FeatureCounts v1.5.0‐p3. The fragments per kilobase of transcript per million mapped reads of each gene were then calculated based on gene length and read counts.

Differential expression analyses between two groups were performed using the DESeq. 2 R package (v1.20.0). Genes with an adjusted *p* value of less than 0.05 were identified as differentially expressed. GO enrichment analysis of these differentially expressed genes was performed using the clusterProfiler R package, in which the gene length bias was corrected. GO terms with a corrected *p* value of less than 0.05 were deemed significantly enriched.

### qPCR

For real‐time qPCR analysis, cDNA was synthesized using HiScript III RT SuperMix (+gDNA wiper) (Vazyme Biotech) and amplified using a QuantiFast SYBR Green PCR Kit (QIAGEN) on a QuantStudio™ 7 Flex Real‐Time PCR System (Life Technologies). Gene expression levels were normalized to those of GAPDH [69]. The candidate genes were amplified by qPCR using primers *MMP‐1*‐F (5ʹ‐GGGGCTTTGATGTACCCTAGC‐3ʹ) and *MMP‐1*‐R (5ʹ‐ TGTCACACGCTTTTGGGG TTT−3ʹ); *MMP‐10*‐F (5ʹ‐ CTCTGGA GTAA TGTCACACCTCT −3ʹ) and *MMP‐10*‐R (5ʹ‐TGTTGGTCCACCTTTCATCTTC−3ʹ)； *MMP‐12*‐F (5ʹ‐CATGAACCGTGAGGATGTT GA‐3ʹ) and *MMP‐12*‐R (5ʹ‐GCAT GGGCTAGGATTCCACC−3ʹ)；*MMP‐13*‐F (5ʹ‐CCAGACTTCACGATGGCATG‐3ʹ) and *MMP‐13*‐R (5ʹ‐GGCATCTCCTCCATAATTTGGC−3ʹ).

## AUTHOR CONTRIBUTIONS

**Jing‐jing Xia:** Conceptualization; investigation; data curation; writing—original draft; writing—review & editing; supervision. **Qian Zhong:** Data curation; validation; writing—original draft; writing—review & editing. **Zhi‐ming Li:** Methodology; data curation; writing—original draft; writing—review & editing. **Qing‐zhen Wei:** Data curation; writing—original draft. **Liu‐yi‐qi Jiang:** Investigation; validation. **Cheng Duan:** Validation. **Hui‐jue Jia:** Resources. **Yi‐mei Tan:** Resources. **Lian‐yi Han:** Resources. **Jean Krutmann:** Supervision. **Jiucun Wang:** Supervision; conceptualization; writing—review & editing. **Xiao Liu:** Conceptualization; supervision; project administration; writing—review & editing.

## CONFLICT OF INTEREST STATEMENT

The authors declare no conflict of interest.

## ETHICS STATEMENT

This study was approved by the Ethics Committee of the School of Life Sciences, Fudan University, China (approval no. 219). All study participants provided written informed consent before participation.

## Supporting information

Figure S1: Associations between age and skin phenotype.
Figure S2: Association between viruses and bacteria.
Figure S3: Heatmap of the Spearman's correlation between the three species and skin phenotype parameters.

## Data Availability

The sequencing data from this study have been deposited in the CNSA (https://db.cngb.org/cnsa/) of CNGBdb with accession number CNP0000635 and NODE (https://www.biosino.org/node/index) with accession number OEP001168. A website (https://db.cngb.org/microbiome/genecatalog/genecatalog/?gene_name=Human%20Skin%20(10.9 M)) has been set up to better visualize the annotation information of the gene catalog and guide researchers who are interested in using our data set and downloading specific sets of data. The data and scripts were saved at https://github.com/ZQ19961021/skin-microbiome. Supplementary materials (figures, graphical abstract, slides, videos, Chinese translated version and update materials) may be found in the online DOI or iMeta Science http://www.imeta.science/imetaomics/.
