# Supplementary material for: Culture dependent and independent approaches reveal the role of specific bacteria in human skin aging
Source: IMetaOmics. 2024 Sep 10;1(2):e26. doi: 10.1002/imo2.26 (PMC12806412; doi:10.1002/imo2.26)
Supplement: Supplementary file 1 — Figure S1: Associations between age and skin phenotype. Figure S2: Association between viruses and bacteria. Figure S3: Heatmap of the Spearman's correlation between the three species and skin phenotype parameters. [file IMO2-1-e26-s001.docx]

Supporting information to

Culture dependent and independent approaches reveal the role of specific bacteria in human skin aging

Running title: Role of specific bacteria in skin aging

Jing-jing Xia^1,2#^, Qian Zhong^1#^, Zhi-ming Li^1#^, Qing-zhen Wei^1#^, Liu-yi-qi Jiang^1^, Cheng Duan^2^, Hui-jue Jia^2^, Yi-mei Tan^3^, Lian-yi Han^2^, Jean Krutmann^4,5^*, Jiu-cun Wang^1,2,5^*, Xiao Liu^6^*

^1^State Key Laboratory of Genetic Engineering, Collaborative Innovation Center for Genetics and Development, School of Life Sciences, Fudan University, Shanghai 200438, China

^2^Greater Bay Area Institute of Precision Medicine (Guangzhou), School of Life Sciences, Fudan University, Guangzhou 511462, China

^3^Department of Skin & Cosmetic Research, Shanghai Skin Disease Hospital, Shanghai 200443, China

^4^IUF - Leibniz Research Institute for Environmental Medicine, Düsseldorf 40225, Germany

^5^Human Phenome Institute, Fudan University, Shanghai 201210, China

^6^Shenzhen International Graduate School, Tsinghua University, Shenzhen 518055, China

^#^These authors contributed equally: Jing-jing Xia, Qian Zhong, Zhi-ming Li, Qing-zhen Wei

*Correspondence: [Jean.Krutmann@IUF-Duesseldorf.de](mailto:Jean.Krutmann@IUF-Duesseldorf.de) (Jean Krutmann), jcwang@fudan.edu.cn (Jiu-cun Wang), liuxiao@sz.tsinghua.edu.cn (Xiao Liu)

**Figure S1.** **Associations between age and skin phenotype.** Results of the Spearman’s correlation between phenotype parameters including age. (A) forehead (FH). (B) cheek (CK). (C) the back of the nose (NS). Blue, negative correlation; Red, positive correlation. The thickness of the line represents the absolute value of the correlation coefficient.

**Figure S2. Association between viruses and bacteria.** (A) Relationship between viral community and bacterial community by Co-inertia analysis (CIA). Colors represent the three anatomical sites. Lines represent sample projections. Square represents the viral community, and circle represents the bacterial community. (B) Spearman correlation between papillomavirus and bacteria. The color bar represents correlation values. (C) Spearman correlation between *Betapapillomavirus 3*, *Staphylococcus virus phiETA*, *Streptococcus phage IPP18*, and age. The color bar represents correlation values. The significance levels in the Spearman correlation are: *, *p* < 0.05; **, *p* < 0.01; ***, *p* < 0.001.

**Figure S3.** Heatmap of the Spearman's correlation between the three species and skin phenotype parameters. Blue, negative correlation; Red, positive correlation. The significance levels in the Spearman correlation are: *, *p* < 0.05; **, *p* < 0.01; ***, *p* < 0.001.
